# Morbid Bronchophony and Bronchial Respiration

**Published:** 1853-06

**Authors:** 


### *Morbid Bronchophony and Bronchial Respiration. Skoda's Theory of Consonance anticipated by an American Physician.*

It is well known to those who give attention to the literature of the physical exploration of the chest, that a new explanation of the mechanism of morbid bronchophony, and the bronchial respiration, has lately been offered by Dr. Joseph Skoda, of Vienna. The explanation of Laennec was, that, the lung being consolidated by effused fibrin or tuberculous deposit, and becoming a better conductor of sound than the spongy structure in its normal condition, the vocal sounds produced by the larynx in bronchophony, and the respiratory sounds in the bronchial tubes are transmitted with greater facility to the ear of the auscultator, the vesicular murmur being at the same time suppressed, the latter, in health, drowning, as it were, the sounds originating in the air tubes. This is the view which has generally been taken by writers on physical exploration, and it is believed to be the opinion now entertained by most practical auscultators.

The theory offered by Dr. Skoda, which is beginning to receive considerable attention, and appears to be gaining favor among writers on the subject in Great Britain and in this country, explains the mechanism of these physical signs on the principal of *Consonance.* This explanation is clearly and concisely set forth in the preface to a translation of Dr. Skoda's Treatise on Auscultation, by Dr. W. O. Markham, of London.* As the readiest mode of presenting it we quote the following extract:

"The voice passes into the parenchyma of the lungs through the medium of the air in the trachea and the bronchial tubes, and is not propagated along their walls; it traverses healthy, as readily as it does hepatized lung, and

---

* The translation has very lately been published, and is not, as yet, reproduced in this country.

even somewhat more readily: consequently, bronchophony does not depend upon an increase of the sound-conducting power of consolidated pulmonary tissue; moreover, when the lung is consolidated, the thoracic voice increases and diminishes in force, without any concurrent change taking place in the condition of the lung: this variation in its strength evidently results from the circumstance of the bronchial tubes being at one moment blocked up by mucus, etc., and at another freed therefrom by the cough and expectoration, etc.: if the bronchophony depended upon conduction of sound, it would be a matter of indifference whether the tubes contained air or fluids. It must not be forgotten, that, according to the ordinary laws of reflection of sound, the more solid the parenchyma the more difficult does the passage of the sound from the air into it become.

"That the air in the mouth and nasal cavities consonates with sounds formed in the larynx, is proved by the fact of the changes which the voice undergoes through opening and closing the mouth and nose, whilst the condition of the larynx remains unaltered; just in the same way does the air in the trachea and bronchial tubes consonate with the laryngeal sounds. Now, air consonates only in a confined space, and the force of the consonance depends upon the form and size of the space, and upon the nature of the walls forming it: the more solid the walls, the more completely will the sound be reflected, and the more forcible the consonance. The cause of the loud voice produced by a speaking-trumpet is well known. But the air will consonate with certain sounds only; in the trachea and bronchial tubes, it becomes consonant with the laryngeal voice, in so far as their walls have a like or an analogous character to the walls of the larynx, of the mouth, and of the nose. Within the cartilaginous walls of the trachea and the bronchial trunks, the voice consonates nearly as forcibly as in the larynx; but as the bronchial tubes divide in the lungs, they lose their cartilaginous character, becoming at last merely membranous in structure, and therefore very ill-adapted for consonance; when, therefore, the consonance is increased in these latter tubes, we may be sure, either that the membrane forming them has become very dense or cartilaginous, or that the tissue around them is condensed and deprived of air, whereby the sound-reflecting power of the tubes is increased. Of course the communication between the air in the tubes and the air in the larynx must be uninterrupted.

"The walls of a confined space frequently vibrate in unison with sounds excited within it, as do those of an organ-pipe, or of a speaking-trumpet. The larynx vibrates with every sound, and its vibrations are perceptible at a considerable distance from their point of origin; so, also, must the walls of the bronchial tubes, which are distributed through the parenchyma of the lungs, vibrate when the voice consonates within them; and the vibrations thus excited will extend to the surface of the thorax, passing through several inches of thick fleshy parts, or of fluids, and manifest themselves there as the consonating sounds of the bronchial tubes."

The theory enunciated in the foregoing quotation, will possess interest for those who are fond of investigating the physical principles involved in the phenomena of auscultation and percussion. We do not propose, at the present time, to discuss the merits of this theory. We are not prepared to do so; and, we are free to confess, we have not formed any opinion respecting its correctness. We have another object in view, in bringing the subject to the notice of our readers. Before proceeding to state this object, however, we would offer a remark to counteract an impression which might naturally be derived, by one not much acquainted with physical exploration, from the existence of discrepancy of opinion among distinguished auscultators as to the mechanism of the physical signs. It is frequently the case that the *pathological* significancy of the phenomena of auscultation and percussion is independent of the explanation of the *physical* principles involved in these phenomena. At all events, this is true in the present instance. Whether *bronchophony and the bronchial respiration* be explained on the theory of conduction of sound exclusively, or the reproduction of sound by consonance, these signs are equally significant of lung consolidation. Their practical import is thus the same in either view. The value of these, and other signs, in diagnosis, is not determined by any *a priori* examination of the physical principles involved in their production, but by ascertaining, by means of observation, during life and after death, with what morbid conditions they are associated so uniformly that they may serve to indicate these conditions. Their significance, in other words, is based on induction and analysis, not on a theoretical synthesis. It is by no means necessary to know the mechanism by which the signs are produced in order to avail ourselves of them as diagnostic guides.

We come now to the chief end we have had in view in writing this article. The theory of consonance was entertained, and publicly enunciated, in this country, many years ago by Dr. Edson Carr, of Canandaigua, N. Y. In an address delivered before the Ontario County Medical Society, at Geneva Medical College, in January, 1841, this explanation was given in brief, but distinct terms, by the gentleman just named. The address just referred to was never printed, nor has Dr. C. taken any pains to publish his views on the subject. Our knowledge of the fact just stated, was accidental. In a conversation with Dr. C., knowing that he had bestowed considerable attention on the physical principles involved in auscultation and percussion, we inquired respecting his opinion of the mechanism of bronchophony and bronchial respiration. We then learned that he had for many years held the doctrine of consonance, which had originated in his own reflections. The address referred to, Dr. C. has placed in our hands, at our request. We take the liberty of quoting a portion, which, although it relates mainly to another point, viz., the identity of bronchophony and pectoriloquy, incidentally embraces the theory of consonance as applicable to all vocal phenomena, and to respiration:

"It is extremely difficult in many instances, to distinguish between morbid bronchophony and pectoriloquy. The fact has been repeatedly noticed by writers on auscultation. I am not aware, however, that its *cause* has ever been explained.

"Why two conditions of the lung, differing so widely in their physical character, should give rise to physical signs so nearly resembling each other that their difference is scarcely appreciable, is a question which very naturally presents itself in this connection.

"Although it is foreign to my present object to enter into a discussion of the principles of acoustic phenomena, on which auscultation is based, yet it may not be unprofitable to dwell a moment on this point.

"If we apply the stethoscope to the larynx of an individual while speaking, we hear not only the sound of the voice, but the distinct articulation of words, as though they were spoken through the instrument. But on removing it to the upper part of the chest, over a large bronchial tube, (if the lung be in a healthy condition,) we perceive a gentle fremitus accompanied by occasional vocal resonance, or bronchophony, whenever the voice in its inflections, passes through certain keys. But there will in this instance be nothing like articulation, most of the vibrations being lost in the nonconducting spongy tissue of the portion of lung situated between the bronchial tube and the solid walls of the chest.

"Now, if the intervening portion of lung be *consolidated*, the effect will the same as that of bringing the tube in direct contact with the solid walls [illegible] the chest, (the latter being a little thickened.) We shall now have *morbid* bronchophony—the vocal sounds being nearly the same as when the stethoscope was applied to the larynx, but with this difference; the articulation of words will be lost in the different media through which the vibrations are conducted before reaching the ear.

"If to this condition of things be added a small cavity near the surface of the consolidated portion of lung, the phenomena afforded, will be distinct pectoriloquy.

"We are told that the most perfect pectoriloquy is produced in cavities of *moderate size*, situated near the surface of the lung, freely communicating with a large bronchial tube—(and by the term *moderate size* I have no doubt is here intended something near that of the larynx.) We shall in this case be presented with two cavities of equal size connected by a large bronchial tube opening into each—(one in the lung, the larynx constituting the other.) Now, is it not quite plain, that whatever vibratory motion, or sound, is produced in one of these cavities, thus situated, will be instantly conducted to, and repeated, or echoed in the other? and over whichever of these the stethoscope be applied, the sound will be the same? If, however, the cavities be of equal size, the echo will be incomplete, and articulation will be lost in the confusion of the unequal vibrations, although the *tones* of the voice will continue unchanged.

"Now it must be borne in mind that the alternate contraction and relaxation of the laryngeal muscles, have the effect of constantly changing the size of the larynx to adapt it to the ever-varying tones of the voice, which, in its inflections, traverses every note of the diapason with the rapidity of thought. Hence it is, that in the most perfect pectoriloquy, although the voice is heard in all its varied tones, we perceive but now and then a distinct word.

"Now without the existence of a cavity in the consolidated portion of lung, if there be a morbid dilatation of one or more bronchial tube, (as not unfrequently happens in the condition of the lung, which we are considering,) it will afford nearly or quite perfect pectoriloquy, on some of the higher keys of the voice—and hence the difficulty which sometimes arises in discriminating between the two conditions.

"The principle on which all these various phenomena may be explained, if fully traced out, is clearly illustrated in the familiar phenomena exhibited by musical instruments, when if one string be struck or caused to vibrate, the corresponding string to every other instrument in the room may be seen to vibrate and echo the same sound. Or if the key to a piano be struck with the sounding board exposed to view, not only the string belonging to that key, but every octave through the whole range, will be seen to vibrate at the same time."

As we have already remarked, we are not prepared to discuss, nor to offer any opinion of the merits of what is generally known as Skoda's theory of consonance. All must admit that it is ingenious and plausible. And whatever credit it may confer we wish to claim in behalf of an American physician, with whom it was not less original than with Skoda, and who has the evidence of having publicly enunciated it before any publications by Skoda had appeared.*

It is unnecessary to remind our readers that the name of Dr. Carr is already identified with the literature of the physical exploration of the chest. The explanation of the mechanism of the *crepitant râlè* submitted by him several years' ago, is the most rational and satisfactory that has ever been offered, and will, as we believe, ultimately supersede all others.

* We make this statement without being positively certain that no work by Skoda was published in 1841, the date of Dr. Carr's address. We presume the statement to be correct.

