# Erratum

**Published:** 1853-06

**Authors:** 


*Erratum.* — On page 56, in the 13th line, for *equal* size, etc., read *unequal.* Article on Skoda's Theory of Consonance.

---

The name of the senator who is entitled to the honor of defeating the anatomical bill in the New York Legislature after it had passed both branches, is Wm. H. Vanschoonhoven, of Troy.

